# Whole body diffusion weighted MRI – a new view of myeloma

**DOI:** 10.1111/bjh.13509

**Published:** 2015-05-26

**Authors:** Christina Messiou, Martin Kaiser

**Affiliations:** ^1^ Department of RadiologyThe Royal Marsden HospitalSurreyUK; ^2^ Division of Molecular PathologyThe Institute of Cancer ResearchSuttonSurreyUK

**Keywords:** myeloma, MRI myeloma, MRI, imaging, functional studies

## Abstract

The recent consensus statement from the International Myeloma Working Group has introduced the role of whole body (WB) magnetic resonance imaging (MRI) into the management pathway for patients with multiple myeloma. The speed, coverage and high sensitivity of WB diffusion weighted (DW)‐MRI and the unique capability to quantify both burden of disease and response to treatment has led to increasing implementation at leading centres worldwide for imaging malignant marrow disease, both primary and metastatic. WB DW‐MRI is likely to have a significant impact on management decisions and pathways for patients with multiple myeloma. This review will introduce the basic principles of DW‐MRI, present current evidence for patients with myeloma and will discuss practicalities and exciting future applications.

The recent consensus statement from the International Myeloma Working Group (IMWG) has recommended whole body (WB) magnetic resonance imaging (MRI) for work‐up of solitary bone plasmacytoma and all patients suspected of having asymptomatic or smouldering multiple myeloma (SMM) (Dimopoulos *et al*, 2015). As per IMWG consensus, patients with more than one lesion >5 mm should be considered as having symptomatic disease requiring therapy whereas previous recommendations recognized only cortical lytic lesions on plain film or computerized tomography (CT) as indicators for treatment (Rajkumar *et al*, 2014; Dimopoulos *et al*, 2015).

Until now MRI of the spine has been performed for patients with SMM; however, a positive MRI in the context of a negative skeletal survey has not consistently been used as an indication to treat (Dimopoulos *et al*, 2009). Emerging evidence indicates that outcomes can be modified by treating patients at high risk of progression from SMM to symptomatic disease. It has been suggested that WB coverage is necessary, as 50% of lesions would be missed by imaging the spine alone (Bauerle *et al*, 2009). Around 16% of patients with SMM will have focal lesions on MRI of the spine (Kastritis *et al*, 2013).

The risk of progression to symptomatic myeloma for patients with SMM is about 8% per year after diagnosis (Kastritis *et al*, 2013). The 2013 landmark study (Mateos *et al*, 2013) demonstrated a potential benefit of early therapy for high risk SMM patients and evidence suggests that MRI can be used as a prognostic biomarker (Moulopoulos *et al*, 1995, 2005; Mariette *et al*, 1999). The Southwestern Oncology Group S0120 study reported that detection of multiple focal lesions on MRI conferred an increased risk of progression (Dhodapkar *et al*, 2015) and an abnormal signal on MRI has been shown to be associated with very high risk of SMM progression and with development of lytic bone lesions (Kastritis *et al*, 2013).

Conventional MRI sequences have superior sensitivity and specificity for disease detection as MRI interrogates marrow in contrast to plain film and CT, which image the secondary effects of myeloma on cortical bone. Although the concept of imaging tumour metabolism with ^18^F‐fluorodeoxyglucose positron emission tomography (^18^F‐FDG PET)/CT is highly appealing, its sensitivity is diminished by poor detection of diffuse marrow infiltration (Zamagni *et al*, 2007; Shortt *et al*, 2009) (Fig 1). As diffuse infiltration in the conventional chemotherapy era has reportedly conferred the worst prognosis compared to focal, variegated or normal patterns, the role of ^18^F‐FDG PET/CT needs careful consideration. Although MRI achieves better results than PET‐CT in staging, the advantage for PET/CT may be speedier changes in response to treatment (Spinnato *et al*, 2012).

**Figure 1:**
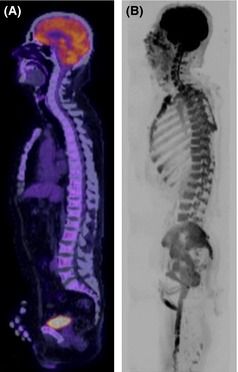
Diffusion weighted magnetic resonance imaging (DW‐MRI) shows increased sensitivity for diffuse marrow infiltration. Sagittal ^18^F‐fluorodeoxyglucose positron emission tomography (^18^F‐FDG PET)/computerized tomography (CT) (A) and b900 whole body DW‐MRI Maximum Intensity Projection image in a 52‐year‐old male with multiple myeloma. (A) ^18^F‐FDG PET/CT was reported as normal with no areas of increased FDG uptake and no lytic lesions on the CT component. (B) Inverted greyscale WB DW‐MRI demonstrated diffuse marrow infiltration. Marrow trephine confirmed 80–90% infiltration with plasma cells.

There have also been attempts to use MRI for response assessment (Giles *et al*, 2014). The sensitivity of cell‐based methods to detect minimal residual disease has significantly improved but combination with a highly sensitive imaging method may overcome the inherent sampling bias of bone marrow biopsy based tests. The MRI pattern has been reported to change in response to therapy, however, nonviable persistent focal lesions may cause an undesirable high rate of false positive findings that can potentially be overcome by WB diffusion weighted (DW)‐MRI.

Despite the advantages of WB MRI the practicalities of delivering such a service are not insignificant. For many centres, this will constitute funding and designing a new clinical service as WB MRI is not yet standard practice for any other malignancy, although a recent European Organization for Research and Treatment of Cancer position statement suggests probable adoption for imaging metastatic bone disease (Lecouvet *et al*, 2014). Currently, the IMWG consensus statement confines the recommendations to use of conventional T1, T2, STIR and post‐contrast sequences with reference made to emerging WB DW‐MRI techniques.

Since the introduction of WB DW‐MRI (Takahara *et al*, 2004), the imaging community quickly recognized its potential for imaging bone marrow. The speed, coverage and high sensitivity of WB DW‐MRI and its unique capability to quantify both burden of disease and response to treatment has led to increasing implementation at leading centres worldwide for imaging malignant marrow disease, both primary and metastatic. This review will introduce the basic principles of DW‐MRI, present current evidence and will discuss practicalities and exciting future applications.

## Basic principles of DW‐MRI

Diffusion weighted‐MRI produces images where the contrast between tissues is based on differences in the motion of water at a cellular level. Water motion in tissues occurs within different compartments – intracellular, transmembrane, extracellular and intravascular. Choice of diffusion weighting (*b* value) influences which compartments are interrogated. Low *b* values interrogate large diffusion distances and are therefore sensitive to flow in blood vessels. Large *b* values detect small diffusion distances that are postulated to be related to extracellular space distances and, therefore, thought to be directly related to cell packing/cellularity. Theoretically, with very large *b* values (>4000 s/mm^2^), it is possible to interrogate the movement of intracellular water protons, but at a practical level this is extremely challenging.

Imaging with more than one *b* value allows automated calculation of an apparent diffusion coefficient (ADC) for each pixel in the image and a quantitative map can be produced. Tumours that consist of tightly packed cells therefore appear as areas of highly restricted diffusion‐high signal on source diffusion images and low on ADC (Messiou & deSouza, 2010). For patients with myeloma it has been shown that *b* values of around 1400 s/mm^2^ are optimal for maximizing contrast between normal and infiltrated marrow (Messiou *et al*, 2011). However there are technical challenges to achieving such high *b* values as the signal to noise is poor, requiring lengthy multiple acquisitions, which makes a WB protocol impractical. Therefore a *b* value of 900 s/mm^2^ is often chosen as a compromise between robust data and WB coverage achievable in a reasonable time frame.

The inverse correlation between cell density in soft tissues and ADC has been extensively described (Sugahara *et al*, 1999; Lyng *et al*, 2000; Guo *et al*, 2002; Tamai *et al*, 2007). This is further supported by indirect evidence that choline, a marker of cell turnover, is inversely correlated with ADC in glioma (Gupta *et al*, 1999). However cellularity is not the sole factor influencing ADC and the relationship between cellularity and ADC has not been so impressive in other cell types (Gupta *et al*, 1999; Guo *et al*, 2002). Other factors may influence ADC, for example, cellular architecture, cell size and size variability within tissue, viscosity of cytoplasm, bulk flow in capillaries and active transport have been variously implicated in small studies. It is likely therefore that ADC is a complex function of tissue microarchitecture that is influenced by several components.

In response to treatment, the increased extracellular spaces within a tumour manifests as increases in distances of water motion and an increase in ADC (Chenevert *et al*, 2000). This has been demonstrated in several tumour types including brain, breast, prostate and liver metastases (Ross *et al*, 1994; Chenevert *et al*, 2000; Theilmann *et al*, 2004; Charles‐Edwards & deSouza, 2006), where it has been used to predict treatment response ahead of conventional imaging and serum markers (Jennings *et al*, 2002; Moffat *et al*, 2006). Some studies have gone further and used DW‐MRI to detect the emergence of drug resistance during the course of therapy (Lee *et al*, 2006).

**Figure 2:**
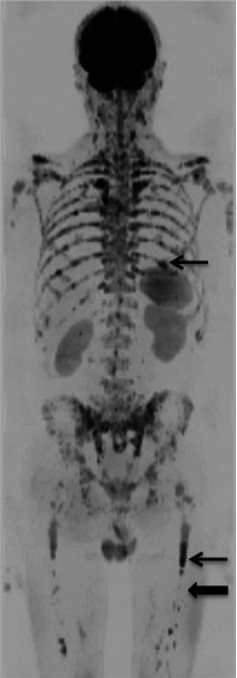
Whole body diffusion weighted magnetic resonance imaging (WB DW‐MRI) provides excellent contrast between normal bone marrow and focal lesions. b900 WB DW‐MRI Maximum Intensity Projection image in a 55‐year‐old male with multiple myeloma demonstrates multiple focal lesions that appear low signal on the inverted grey scale image (examples indicated by arrows) with excellent contrast against normal marrow, which does not return signal (block arrow).

However the presence of fat in marrow necessitates an adapted approach. In normal adult marrow, fat predominates and there is a paucity of free water. The motion of the small amount of water that is present is restricted by fat and hence normal adult marrow has very little signal on DW‐MRI. As cellularity in marrow increases secondary either to disease or increased haematopoietic tissue, the amount of free water increases and so does ADC (Nonomura *et al*, 2001; Hillengass *et al*, 2010). It is thought that the increased vascularity associated with plasma cell infiltration is also influential in this relationship (Hillengass *et al*, 2010). Hence, in adults the different microarchitecture of plasma cell infiltrated marrow results in markedly different signal on DW‐MRI and contrasts against normal marrow, which returns little signal (Fig 2).

## Whole body DW‐MRI in myeloma

### Detection

The excellent image contrast between normal and diseased marrow on WBDW‐MRI results in superior lesion conspicuity compared to conventional short tau inversion recovery and contrast‐enhanced MRI sequences (Pearce *et al*, 2012; Dutoit *et al*, 2014; Squillaci *et al*, 2014). Furthermore, unlike extra skeletal tumour types, the ADC value of myeloma‐infiltrated marrow is significantly different to normal adult marrow with very little overlap (Hillengass *et al*, 2011; Messiou *et al*, 2011). This means that qualitative differences in image contrast translate into quantitative differences and the ADC value can be used to separate myelomatous from normal marrow with a sensitivity of 90% and specificity of 93%. The ADC of marrow of patients in remission and with monoclonal gammopathy of undetermined significance is not significantly different to normal age‐matched volunteers, making WB DW‐MRI a promising tool for monitoring these patients (Messiou *et al*, 2011). We should note however that the new IMWG guidelines stipulate focal lesions only as an indication to treat in patients with asymptomatic myeloma despite the poor prognosis associated with diffuse infiltration (Dimopoulos *et al*, 2015). This is perhaps because diagnosis of diffuse infiltration on conventional MRI is challenging and is most often a subjective diagnosis based on comparison of marrow signal with intervertebral discs. There is potential for ADC measurements to reduce this subjectivity and this should be a priority for future studies. However accurate representation of trephines in patients with diffuse infiltration is more likely compared to patients with multifocal disease.

Although WB DW‐MRI is emerging as one of the most sensitive tools for imaging bone marrow (Wu *et al*, 2011; Lecouvet *et al*, 2012; Pearce *et al*, 2012), some debate remains as to its specificity. Although Lecouvet *et al* (2012) presented data to suggest high specificity for detection of metastatic bone disease (98–100%), a recent meta‐analysis showed a pooled specificity of 86·1% (Wu *et al*, 2011). The paucity of myeloma‐specific prospective studies and marked heterogeneity in reference standards make current judgments on specificity challenging and biopsy of all lesions is not feasible. The approach offered by the IMWG of 3‐ to 6‐month follow‐up of equivocal solitary small lesions is a pragmatic solution.

**Figure 3:**
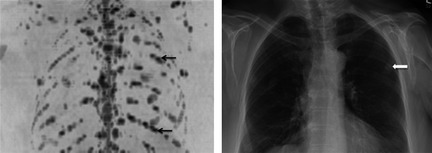
Diffusion weighted magnetic resonance imaging (DW‐MRI) is highly sensitive for detection of focal rib lesions. Left: Inverted greyscale b900 whole body DW‐MRI Maximum Intensity Projection of the chest in a 53‐year‐old female with multiple myeloma shows numerous sites of disease in the ribs (examples indicated by arrows). Right: A chest radiograph in the same patient shows rib fractures (block arrow) but no lytic lesions.

The skull and ribs have historically been difficult sites for interrogation with MRI, however DW‐imaging has shown increased sensitivity for lesion detection in the ribs compared to skeletal survey in 2 studies (Narquin *et al*, 2013; Giles *et al*, 2015) (Fig 3). However lesion detection in the skull was reduced compared to skeletal survey in both studies. This is possibly because the small volume of marrow in the skull is challenging to interrogate against adjacent high diffusion signal in the brain. However, false positive results on plain film of the skull secondary to venous lakes and granulations are also possible and difficult to confirm.

### Assessment of disease status and response to treatment

**Figure 4:**
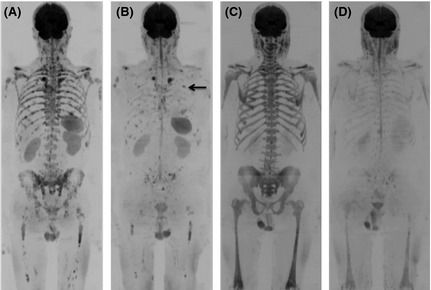
Subjective assessment of response on whole body diffusion weighted magnetic resonance imaging (WB DW‐MRI). Inverted greyscale b900 WB DW‐MRI Maximum Intensity Projection pre‐ (A) and 98 d post‐CVD (cyclophosphamide, bortezomib and dexamethasone) (B) in a 63‐year‐old male with multiple myeloma demonstrates multiple focal lesions reducing in size and number. Small volume sites of disease persist (B, example shown by arrow). International Myeloma Working Group (IMWG) response status was very good partial response. Inverted greyscale b900 WB DW‐MRI Maximum Intensity Projection pre‐ (C) and 92 d post‐DT‐PACE (dexamethasone, thalidomide, cisplatin, doxorubicin, cyclophosphamide and etoposide) (D) in a 67‐year‐old male with multiple myeloma demonstrates diffuse marrow infiltration which normalizes after treatment. IMWG response status was complete response.

The capability of WB DW‐MRI to demonstrate both focal and diffuse marrow infiltration throughout the whole skeleton makes this extremely promising as a subjective tool for monitoring disease status and assessment of response. For focal lesions, changes in size and number can be easily assessed and for diffuse infiltration, signal changes are evident (Fig 4). However, ADC measurements offer the capability to quantify disease throughout the skeleton. Advances in data informatics have made semi‐automated skeletal segmentation a reality, which enables histogram quantification of a patient's whole marrow. This has been demonstrated by Giles *et al* (2014) who used these techniques to segment patients bone marrow on DW‐MRI to quantify response to treatment. Reassuringly, the reproducibility was excellent with a coefficient of variation of 2·8%. Mean ADC increased in 95% of responding patients and decreased in all non‐responders (*P* < 0·002). A 3·3% increase in ADC helped identify response with 90% sensitivity and 100% specificity. Visual assessment was also able to identify response to treatment with high sensitivity (sensitivity 86%, specificity 95%) with good agreement in the post‐treatment change between 2 observers. There was a significant negative correlation between change in ADC and change in laboratory markers of response. Conversely, Hillengass *et al* (2011) demonstrated a decrease in ADC following therapy and it is likely that the direction of ADC change is influenced by the timing of the measurement. Messiou *et al* (2012) have confirmed that early following treatment ADC increases, presumably due to plasma cell death, and resultant increased extracellular spaces and later follow up measurements show an ADC fall when normal marrow architecture including marrow fat are restored.

### Prognosis

**Figure 5:**
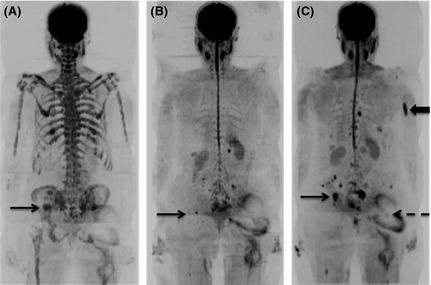
The extremely high sensitivity of diffusion weighted magnetic resonance imaging (DW‐MRI) allows detection of tiny foci of residual disease post‐autograft. b900 whole body DW‐MRI Maximum Intensity Projection images of a 70‐year‐old female with multiple myeloma before (A), 3 months post‐ (B) and 8 months post‐ (C) autograft. At baseline there is widespread marrow disease including a focal lesion in the right iliac bone (A, arrow). At 3 months post‐autograft, the marrow signal has normalized apart from a tiny residual focus of abnormal signal at a site of original disease in the right iliac bone (B, arrow). WB DW‐MRI 8 months post‐autograft shows progression of the residual disease (C, arrow) and development of new sites (example shown by block arrow). Ill‐defined distortion overlying the left hip (dashed arrow) represents artefact from a metal hip prosthesis.

Compelling data from Bartel *et al* (2009) and Zamagni *et al* (2011) have shown 18‐F FDG PET/CT to be prognostic in the post‐induction and post‐transplant phases, respectively. Accurate prognostic information in these settings, where treatment can be both costly and associated with toxicity, is highly desirable for patient selection but also as a tool to stratify treatment intensity, consolidation or maintenance therapy. Hillengass *et al* (2012) performed conventional WB MRI in patients before and after single or double autologous stem cell transplantation (ASCT) treatment and found that the number of detected focal lesions on MRI significantly correlates with and predicts overall survival in patients. Whilst FDG PET/CT has been shown to be a powerful tool in detecting residual disease, the increased sensitivity of DW‐MRI and the capability to detect both tiny deposits and diffuse disease is likely to give it an advantage that merits prospective evaluation (Fig 5).

## Clinical WB DW‐MRI

### Whole body MRI protocol

**Figure 6:**
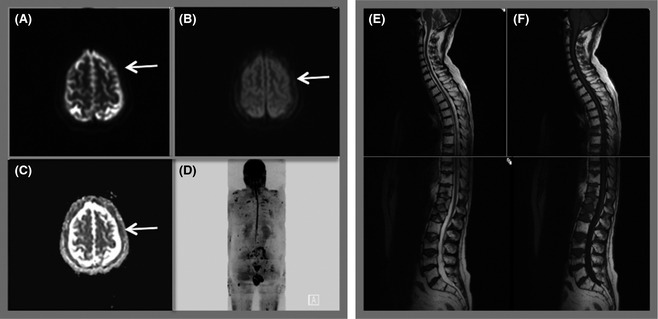
Whole body MRI protocol. Diffusion weighted magnetic resonance imaging (DW‐MRI) is performed axially using *b* values of 50 (A) and 900 (B). The system software automatically generates an apparent diffusion coefficient map (C) for each slice using the *b* 50 and 900 data. Axial images through the head demonstrate abnormal signal in the skull vault indicating infiltration (arrows). Radiographers post‐process the data to produce the 3‐dimensional inverse grey scale Maximum Intensity Projection image (d). Whole body DW‐MRI is supplemented by sagittal T2 (e) and T1 (f) weighted images of the spine. Total imaging time 45 min.

Developing a WB DW‐MRI protocol necessitates balancing robust data acquisition that fulfills clinical need and a time frame that is tolerable for the patient (Fig 6). Currently WB DW‐MRI is best achieved in the axial plane. At our institution, *b* values of 50 and 900 s/mm^2^ are used with coverage from the skull vertex to the knees and automated software generates an ADC map for each axial slice. Radiographers post‐process the *b* 900 s/mm^2^ data to produce a 3‐dimensional image of the skeleton. Although the DW data provides remarkable images of bone marrow, anatomical detail is limited and therefore we supplement the protocol with a ‘fast’ T1weighted (W) and T2W sagittal of the whole spine. This allows assessment of vertebral fractures and threat to the spinal cord or nerve roots. However, a total imaging time of 45 min necessitates appropriate patient selection. For example if a patient presents with symptoms suspicious for spinal cord compression this scanning time may not be tolerable and imaging should be confined to assessment of disease in the spinal column. Referring haematologists and specialist myeloma nurses at our institution have become well acquainted with imaging times and refer patients appropriately and therefore WB protocols are extremely well tolerated at our institution.

The combination of morphological and functional data also allows for detection and characterization of vertebral fractures. Although morphological features of diffuse T1 low signal, convex vertebral contour, involvement of pedicles and a lumbar level are more frequently observed in malignant fractures (Moulopoulos *et al*, 1996) the addition of DW‐MRI to conventional MRI sequences has been shown to improve diagnostic accuracy (Sung *et al*, 2014). Theoretically benign fractures have more oedema and free water and therefore a higher ADC than malignant fractures, where packed tumour cells lower the ADC. However, there has been conflict in the literature as to whether ADC can reliably be used as a sole discriminator (Geith *et al*, 2012, 2014; Sung *et al*, 2014). It seems likely that fracture age and mixed oedema and tumour are likely to contribute to overlap in ADC values of benign *versus* malignant fracture. Greater understanding of appropriate diffusion protocols and combination with morphological imaging is likely to move this forward as a useful tool.

The diffusion sequences are also exquisitely sensitive to trephine tracts and if this has already been performed, WB DW‐MRI can give some indication of the representation of the trephine sample. Alternatively, the WB DW‐MRI can be used to select the side for trephine sampling.

### Incidental findings

The high sensitivity of WB DW‐MRI in bone marrow and soft tissues will undoubtedly lead to detection of incidental findings and these are common in WB DW‐MRI examinations, but only a minority are equivocal and require further action (Lecouvet *et al*, 2012). The use of WB MRI in patients with myeloma is likely to increase and hence we will need to develop strategies to manage incidental findings. The reporting radiologist should facilitate this by specifically expressing a level of concern and making suggestions regarding the need for further investigations. In our experience WB MRI also increases detection of asymptomatic extramedullary myeloma, changing our perspective of the disease. WB DW‐MRI is also sensitive to changes in the femoral heads that indicate avascular necrosis, which is not uncommon in patients with myeloma treated with corticosteroids.

### Clinical indications

**Figure 7:**
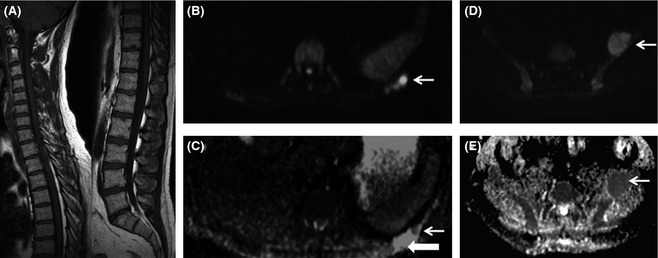
Whole body diffusion weighted magnetic resonance imaging (WB DW‐MRI) can differentiate active from inactive sites and reveals unexpected additional sites of disease. A 53‐year‐old female with a previous history of solitary rib plasmacytoma treated with radiotherapy, with rising paraproteins. Weighted T1 (T1W) MRI of the spine (A) was normal. WB DW‐MRI [axial b900 B, apparent diffusion coefficient (ADC) map, C] demonstrated a 3 mm focal nodule of restricted diffusion (arrows, B and C) within the treated rib bone defect (block arrow, C), in keeping with local recurrence. In addition WB DW‐MRI showed a 5 cm expansile focus of disease arising from the left iliac bone [arrows on b900 (D) and ADC map (E)].

The most recent consensus statement from the IMWG (Dimopoulos *et al*, 2015) recommends WB MRI in the workup of solitary bone plasmacytoma and patients with SMM. We have extended the service to monitoring patients with non‐secretory myeloma. On a case‐by‐case basis it is also used to guide therapeutic decisions and to monitor remission status in patients with symptomatic clinically or genetically defined high risk disease. For patients with previously treated myeloma with borderline remission status or possible recurrence, WB‐DWI is also extremely useful in differentiating treated from active sites of disease to guide management (Fig 7).

### Pitfalls

**Figure 8:**
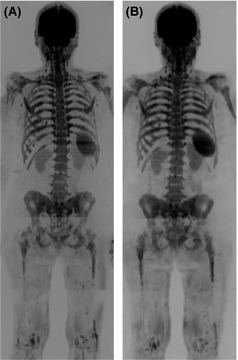
False positive whole body diffusion weighted magnetic resonance imaging (WB DW‐MRI) following granulocyte colony‐stimulating factor (GCSF) administration. b900 WB DW‐MRI Maximum Intensity Projection images in a 67‐year‐old female with relapsed multiple myeloma (A) show diffuse abnormal marrow signal indicating infiltration which was confirmed by 20% plasma cell infiltration on trephine. b900 WB DW‐MRI Maximum Intensity Projection images 6 months later following treatment (B) show stable appearances; however; trephine showed regenerating marrow and no plasma cells. The patients had received GCSF 3 d prior to the MRI (B), which caused a false positive MRI secondary to marrow hypercellularity.

Red marrow has an altered diffusion signal compared to yellow marrow and therefore detection of marrow disease in younger patients can be challenging. However this is rarely problematic for myeloma as the incidence is strongly related to age with an average of 43% cases diagnosed in people aged 75 years and over (http://www.cancerresearchuk.org/cancer-info/cancerstats/keyfacts/myeloma/). Granulocyte colony‐stimulating factor (GCSF), given either as part of preparation for ASCT or for supportive measures, leads to hypercellular marrow, which can mimic diffuse infiltration (Fig 8). This is also problematic for other imaging techniques such as conventional MRI sequences and 18F‐FDG PET/CT. Therefore DW‐MRI should be avoided in the days following GCSF administration.

## Future directions and conclusion

The advent of WB DW‐MRI has been one of the most significant advances for imaging in oncology patients over the last decade. The high sensitivity, speed and quantitative capabilities have addressed an unmet need in imaging primary and secondary marrow malignancies. We have been providing a WB DW‐MRI service for patients with myeloma since 2011 and the number of UK and International centres delivering this service is growing.

Combined with anatomical imaging, WB DW‐MRI now forms an imaging tool that has been tailored to detect both focal and diffuse disease, to quantify burden and response whilst also assessing mechanical complications. This blend of anatomical and functional imaging is well suited to serve the imaging needs of patients with myeloma. The necessity for WB imaging of patients with SMM and solitary plasmacytoma is now recognized. However future studies will need to direct positioning of WB DW‐MRI alongside emerging molecular and genetic signals as predictive and prognostic biomarkers. If future studies corroborate the usefulness of whole marrow segmentation techniques, academic and industrial collaborations will be essential to translate this from research to clinical settings. It is also likely that WB DW‐MRI will have an increasing role in stratifying risk and, as the number of treatment options continues to grow, it is likely to find its place in guiding therapeutic strategies.

## Author contributions

Both authors have contributed to the concept, literature review and writing of this review article.
